# Evaluation of GSMaP and MSWEP precipitation products for runoff simulation in the Lhasa River Basin

**DOI:** 10.1371/journal.pone.0342995

**Published:** 2026-02-23

**Authors:** Lingling Wu, Yufeng Ren, Peng Huang, Shiyu Yuan, Chun Zhou, Zhongshun Gu, Yinan Guo, Li Zhou

**Affiliations:** 1 Sichuan Hydrological and Water Resources Survey Center, Chengdu, China; 2 China Yangtze Power Co., Ltd, Yichang, Hubei, China; 3 Sichuan Road and Waterway Construction Engineering Co., Ltd, Chengdu, China; 4 State Key Laboratory of Simulation and Regulation of Water Cycle in River Basin, China Institute of Water Resources and Hydropower Research, Beijing, China; 5 State Key Laboratory of Hydraulics and Mountain River Engineering, College of Water Resource & Hydropower, Sichuan University, Chengdu, China; 6 Xizang Autonomous Region Meteorological Information and Network Centre, Xizang, China; 7 Xigazê National Climatological Observatory, China Meteorological Administration, Shigatse, China; 8 National Meteorological Information Center, Beijing, China; 9 Institute for Disaster Management and Reconstruction, Sichuan University, Chengdu, China; National Cheng Kung University, TAIWAN

## Abstract

As the Asian Water Tower, the Tibetan Plateau (TP) plays a pivotal role in regulating the regional water cycle, yet sparse rain gauge networks hinder reliable hydrological modeling and climate change assessments. This study focuses on the Lhasa River Basin, a representative high-altitude basin in central TP, to evaluate the performance of two satellite-based precipitation products, GSMaP and MSWEP, for runoff simulation using the BTOP distributed hydrological model. Precipitation accuracy was assessed using the critical success index (CSI), correlation coefficient (CC), and coefficient of determination (R^2^), while hydrological simulations were evaluated through calibration and validation against observed discharge using the Nash–Sutcliffe Efficiency (NSE). The main findings are: (1) GSMaP generally outperforms MSWEP, with an average CSI of 0.8126 compared to 0.7521, ranging from 0.8690 at Lhasa station to 0.7453 at Biruo station; (2) GSMaP shows stronger consistency with observations, with higher CC and R^2^ values (R^2^ = 0.82 vs. 0.77); (3) both datasets perform well in the wet season (CC > 0.8) but degrade in the dry season, with GSMaP exhibiting smaller time-series errors; and (4) the BTOP model driven by GSMaP achieves higher NSE values in both calibration (0.793) and validation (0.541) periods, more effectively reproducing basin hydrological processes despite some peak flow deviations during flood events. Overall, the results indicate that GSMaP provides more accurate precipitation estimates and runoff simulations in the Lhasa River Basin, offering reliable data support for hydrological research and water resource management in data-scarce regions of the TP.

## Introduction

Precipitation is a key component in the global water and energy cycles, playing a crucial role in hydrology, meteorology, and related disciplines [1,2]. Accurate precipitation and runoff data are vital for disaster forecasting, agricultural planning, rainfall prediction, and scientific research [3,4]. Currently, there are three primary methods for measuring precipitation: rain gauges, weather radar, and satellite sensors [5]. Among them, rain gauges provide the highest accuracy; however, due to the complex terrain and high costs, rain gauge networks are sparse over the Tibetan Plateau. Moreover, conventional ground-based precipitation observations often fail to capture the full spatiotemporal variability of precipitation, limiting their applicability in hydrometeorological studies [6,7]. Weather radars can provide large-scale precipitation information with high spatial and temporal resolution [8]. Nevertheless, ground clutter and terrain blockage can lead to blind spots in low-level precipitation detection. Additionally, radar systems are predominantly deployed in plains and densely populated regions, resulting in limited coverage and reduced accuracy in complex mountainous terrains like the Tibetan Plateau [9,10].

In contrast, satellite-based precipitation products can offer continuous, high-resolution precipitation estimates on a global scale [11–13]. These products have shown significant value, particularly in data-sparse or inaccessible regions. Currently, several mainstream satellite precipitation products, including GPM-IMERG, GSMaP, TRMM 3B42/3B43, CMORPH, PERSIANN, CHIRPS, and MSWEP, are widely applied in hydrological and meteorological research, disaster monitoring, and climate analysis [14–17]. These datasets offer advantages including wide spatial coverage, high spatiotemporal resolution, and near real-time availability. Their utility is especially prominent in regions with limited ground-based observations, such as oceans, mountainous areas, and high-latitude zones [17–19]. However, differences in precipitation retrieval algorithms, data integration strategies, and bias correction approaches among these products lead to substantial spatial and temporal variability in their accuracy across different climate zones, terrains, and precipitation regimes [20]. For instance, IMERG demonstrates relatively high accuracy in low- to mid-latitude regions due to its integration of multiple data sources and ground-based corrections, while GSMaP is better at capturing short-duration precipitation events but tends to have larger errors under complex terrain and heavy rainfall conditions [21]. MSWEP, on the other hand, is a merged product that combines satellite, reanalysis, and gauge data, aiming to provide a globally consistent, long-term precipitation dataset with improved accuracy in both gauged and ungauged regions [22–24]. Furthermore, both products have been applied in various hydrometeorological studies in China and globally, demonstrating relevance to the current study context [25,26].

To improve precipitation estimation accuracy, integrating multiple satellite precipitation products by leveraging their respective strengths and minimizing systematic errors has become a prominent research direction [27]. In recent years, multi-source precipitation data fusion has been increasingly applied in various regions to enhance satellite-based precipitation accuracy [28]. Li et al [29] developed a Transformer-based multi-source precipitation fusion model incorporating environmental factors in the Jingle Basin. The fused data significantly improved rainfall-runoff simulations in the Xin’anjiang, LSTM, and Prophet models, enhancing correlation with observations compared to single-source products. Shi et al [30] proposed an EDGWR framework that integrates satellite and gauge precipitation data, demonstrating superior performance in the Yellow River Source Region. The IMERG-EDGWR product showed particular improvements in heavy precipitation detection and winter estimates, maintaining robustness with limited input data.

With the intensification of global climate change and human activities, the degree of development in the Lhasa River Basin, Tibet, has been steadily increasing, leading to an urgent demand for high-quality precipitation data [31]. Although ground-based meteorological stations in the basin provide relatively accurate precipitation observations, the limited number and uneven spatial distribution of these stations hinder the comprehensive characterization of spatiotemporal variability in precipitation [32,33]. Furthermore, the scarcity of reliable precipitation and runoff observations introduces considerable uncertainty in runoff simulation and water resource forecasting within the basin. Satellite-based precipitation products, with their large-scale continuous coverage and high spatial-temporal resolution, have demonstrated significant potential for application in mountainous and data-scarce regions. However, due to the complex topography and the influence of extreme weather conditions, single-source satellite precipitation products often exhibit considerable estimation errors over the Tibetan Plateau [34]. Discrepancies among different products are observed in terms of precipitation intensity, temporal dynamics, and spatial distribution [35]. These climate-induced changes have further complicated the spatial and temporal variability of precipitation in the region, increasing the demand for accurate and robust precipitation datasets. As such, understanding and reducing uncertainties in satellite precipitation estimates under changing climate conditions is crucial for improving hydrological modeling and supporting climate-resilient water resource management [36].

Therefore, systematically evaluating the accuracy and hydrological utility of satellite precipitation products in the Lhasa River Basin has substantial scientific and practical value. Previous studies over the Tibetan Plateau have mostly focused on either the statistical evaluation of individual SPPs or their application in conceptual hydrological models, whereas comprehensive assessments that jointly consider detection skill, intensity and spatial patterns, and their impacts on physically based distributed runoff simulations remain limited in glacier- and snow-influenced basins in central TP [37]. In particular, few studies have simultaneously compared GSMaP and MSWEP as forcing for a distributed model such as BTOP, and even fewer have examined the temporal transferability between calibration and validation periods in a data-scarce alpine catchment [38]. Based on this rationale, the present study aims to (i) evaluate the detection capability, temporal correlation, and spatial distribution of GSMaP and MSWEP precipitation against gauge observations in the Lhasa River Basin, and (ii) quantify how their differences affect runoff simulations by the BTOP model during a calibration period (2010–2013) and a validation period (2014–2015). By conducting a comparative analysis of runoff simulation accuracy using gauge-based and satellite-based precipitation inputs, this study seeks to identify the more suitable product for hydrological applications in the Lhasa River Basin and to provide guidance for the future design of bias-correction and multi-source fusion strategies in data-scarce high-altitude regions.

## Study area and data acquisition

### Study area

**Fig 1:**
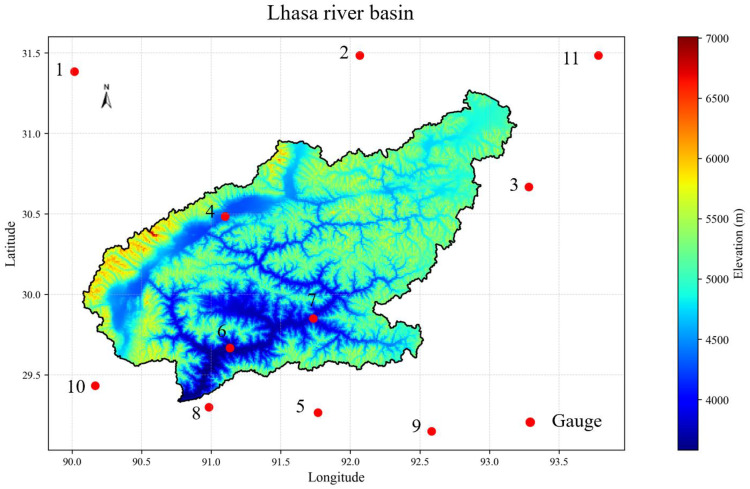
Study area and location of precipitation gauge stations.

The Lhasa River Basin is characterized by abundant water resources, with a multi-year average discharge of 288 m^3^/s and an annual total runoff volume of approximately 9.082 billion m^3^ [39]. The basin spans a total length of 568 km and receives an average annual precipitation of 545.5 mm. An overview of the Lhasa River Basin is illustrated in Fig 1. Although the basin possesses substantial water reserves, both the utilization efficiency and development degree of water resources remain relatively low [40]. As such, the region still holds significant potential for future water resource development. Climatically, the Lhasa River Basin falls within a temperate, semi-arid monsoon regime typical of the Tibetan Plateau, with a low multi-year average temperature ranging from 1.2 °C to 7.5 °C [41]. The elevation data used in this study were obtained from the Shuttle Radar Topography Mission (SRTM) digital elevation model (DEM), with a spatial resolution of 90 meters (version 4.1), provided by the Consortium for Spatial Information (CGIAR-CSI).

### Data acquisition

#### GSMaP dataset.

The Gauge-adjusted Global Satellite Mapping of Precipitation (GSMaP) is a blended microwave and infrared precipitation product from the GPM mission [42]. The GSMaP-Gauge product improves retrieval accuracy by incorporating the CPC unified gauge-based global daily precipitation analysis, with a latency of 3 days [43]. In this study, we adopted the near-real-time gauge-adjusted GSMaP GNRT dataset (version 06), which provides hourly precipitation at a spatial resolution of 0.1° × 0.1° on a global grid. Daily precipitation was obtained by aggregating the hourly data from 00:00–24:00 local time. The analysis period spans from January 1, 2010, to December 31, 2015.

#### MSWEP dataset.

The Multi-Source Weighted-Ensemble Precipitation (MSWEP) dataset, recently developed by Beck et al. (https://www.gloh2o.org/mswep/), is a globally available precipitation product that integrates observations from ground-based rain gauges, satellite remote sensing, and reanalysis datasets. Additionally, the dataset is calibrated using runoff and potential evapotranspiration data from selected river basins to improve accuracy [44]. The original data were provided at a 3-hourly temporal resolution and a 0.1° × 0.1° spatial resolution. To facilitate analysis at different timescales, the 3-hourly precipitation data were aggregated into daily and monthly totals as needed [23]. For the purposes of runoff simulation and evaluation, the daily precipitation data from January 1, 2010, to December 31, 2015, were employed in this study.

#### Rain gauge dataset.

This study also utilized observational data from three hydrometeorological stations located within the Lhasa River Basin. The dataset spans from January 1, 2010, to December 31, 2015, and includes daily measurements of precipitation, air temperature, wind speed, and vapor pressure, among other standard meteorological variables. These ground-based observations were obtained from the China Meteorological Administration’s National Meteorological Information Center (http://data.cma.cn). Although only three meteorological stations are available within this vast and topographically complex region, their spatial distribution, which covers the upper, middle, and lower reaches of the basin, ensures a reasonable representation of the basin’s hydrometeorological and altitudinal gradients (Fig 1). Due to the high elevation, harsh climate, and logistical challenges of the Tibet Plateau, the overall density of observation sites remains sparse. Therefore, while the limited number of stations may impose some spatial limitations, they nonetheless provide the best available ground truth for evaluating satellite-based precipitation products in this data-scarce region.

### Methodology

#### Hydrological model.

**Fig 2:**
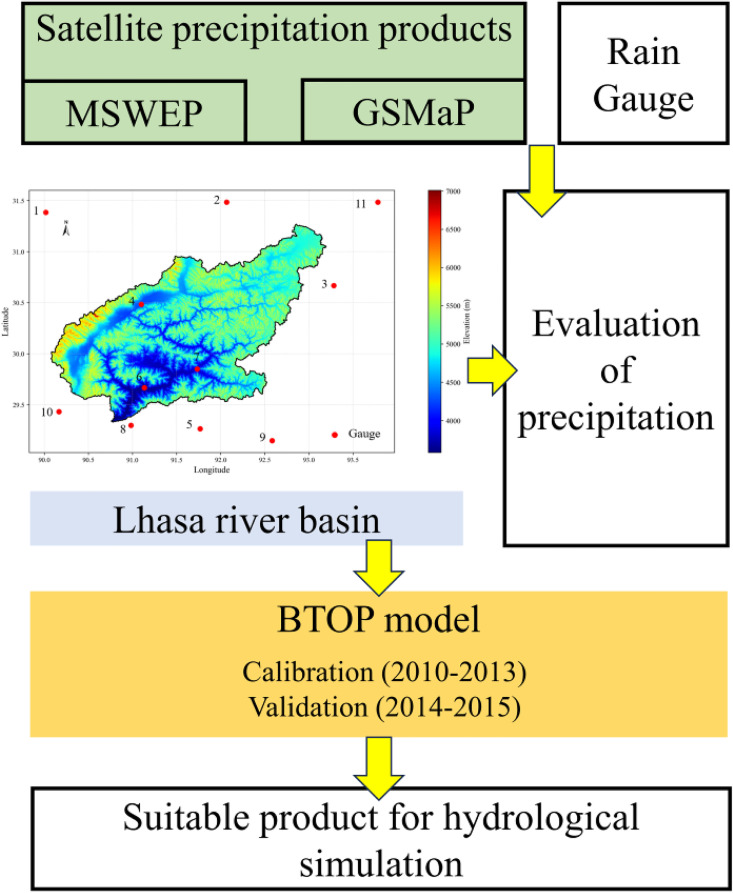
Methodology framework.

Fig 2 presents the overall methodological framework, which includes precipitation evaluation using rain-gauge observations, BTOP hydrological simulations driven by GSMaP and MSWEP, and the final identification of the most suitable satellite product for hydrological application in the Lhasa River Basin.

This study employed the Block-wise TOPMODEL (BTOP), a semi-distributed hydrological model with minimal calibration requirements, combining TOPMODEL’s runoff generation concept [45] and the Muskingum-Cunge routing method [46]. The model’s physically interpretable parameters, derived from soil properties, land cover, and moisture conditions, allow integration with remote sensing and geospatial datasets without extensive ground observations. Moreover, the BTOP model is developed and integrated several modules such as the core runoff generation (TOPMODEL), parameterization (SCE-UA), flow routing (Muskingum-Cunge) and evapotranspiration (S-W) modules, optional snow process, soil freezing, and dam/reservoir modules [47,48]. The parameters and related variables used in the BTOP model are summarized in Table 1. To date, the BTOP model has been widely applied for the flood and drought evaluation, water infrastructure, land-use planning, pollutant transport, and climate variability scenarios in nearly 2300 basins worldwide [49,50].

**Table 1:** Parameters and related variables of the BTOP model.

Parameters	Unit	Physical Interpretation	Range	Mark
*D*_*0clay*_	m·Δt ⁻ ¹	Groundwater dischargebility (Clay)	[0.01, 2]	A1
*D*_*0sand*_	m·Δt ⁻ ¹	Groundwater dischargebility (Sand)	[0.01, 2]	A2
*D*_*0silt*_	m·Δt ⁻ ¹	Groundwater dischargebility (Silt)	[0.01, 2]	A3
*SD*_*bar*_	m	Block-average saturation deficit	[0.001, 0.9]	Bi
*m*	m	Discharge decay factor	[0.01, 0.1]	Ci
*n0*	s·m ⁻ ¹⁄³	Block-average Manning coefficient	[0.00001, 0.8]	Di
*α*	–	Drying function parameter	[–10, 10]	Ei

Note: i = 1,2, …, *n*_*k*_ (*n*_*k*_ is the number of the divided sub-basins).

Required inputs include topography (SRTM DEM, 3 arcseconds), land cover (MODIS-IGBP, 500 m), soil data (FAO, 1:5M), vegetation indices (NOAA NDVI, 0.05°), and meteorological variables (CRU, 0.25°), all resampled to 1 km resolution. Discharge data from Kitamatsuno station (MLIT) were used for validation.

### Model parameterization

In order to ensure the physical rationality and accuracy of the BTOP model in spatial distribution and runoff-routing simulation, this study systematically designed the model from two aspects: parameter calibration and elevation zoning strategy. The model parameters were calibrated using the Shuffled Complex Evolution algorithm (SCE-UA), a widely used global optimization method in hydrological modeling known for its strong search capabilities and convergence performance [51]. The maximum number of iterations was set to 50000 to balance convergence and computational efficiency. The primary objective function was the Nash-Sutcliffe Efficiency (NSE), supplemented by Root Mean Square Error (RMSE) and Bias to comprehensively evaluate the agreement between simulated and observed streamflow. To represent the spatial heterogeneity of hydrological processes affected by terrain, such as precipitation, evapotranspiration, and snowmelt, elevation zoning was applied in the semi-distributed BTOP model [45]. Unlike traditional approaches that divide the watershed into elevation zones, BTOP does not require explicit elevation zoning; instead, elevation influences hydrological processes continuously across the grid domain. This approach ensures a more spatially detailed and physically representative simulation of hydrological dynamics in high-altitude mountainous regions [52].

In this study, the snowmelt process was represented using a temperature-index (degree-day) method, which is widely applied in cold region hydrological modeling due to its simplicity and reliable performance under data-scarce conditions. The snowmelt rate *M* is calculated as:


$$M=DDF\times (T_{a}-T_{melt}),T_{a}>T_{melt}$$

Where: M is the snowmelt rate (mm/day); DDF is the degree-day factor (mm/°C/day); T_a_ is the air temperature (°C); T_melt_ is the melt threshold temperature (typically 0°C). This formulation assumes a linear relationship between air temperature and melt rate, reflecting the dominant influence of sensible heat on snow and ice melt in alpine regions. The degree-day method has been validated in numerous studies over glacierized basins.

Although BTOP does not explicitly simulate glacier mass balance or ice dynamics, its snowmelt module captures the essential seasonal variability of meltwater input by applying the temperature-index approach uniformly to both seasonal snowpack and glacier-covered areas. This provides a reasonable approximation of meltwater contributions during dry periods in high-altitude basins.

For all hydrological simulations in this study, the BTOP model was calibrated and validated using daily discharge at the Lhasa hydrological station over the period 1 January 2010 to 31 December 2015. Consistent with the precipitation analysis, the time series was split into a calibration period (1 January 2010–31 December 2013) and a validation period (1 January 2014–31 December 2015). This split was used consistently in the scatterplot regressions between satellite and gauge precipitation, as well as in the evaluation of BTOP runoff simulations, ensuring that all statistical analyses refer to the same calibration/validation periods throughout the paper.

#### Evaluation metrics.

Qualitative statistical indices were employed to evaluate satellite products, including Pearson correlation coefficient (CC) and Mean Absolute Error (MAE). CC describes the degree of linear correlation between the satellite products; Besides, three categorical indices were adopted to evaluate the detectability of satellite products [53]. The probability of detection (POD) measures the ratio of precipitation events correctly detected by the satellite products. False alarm ratio (FAR) measures the ratio of the no-rain events incorrectly detected by the satellite products. Critical Success Index (CSI) comprehensively reflects the overall fraction of precipitation events correctly detected [54]. NSE measures how well the simulated streamflow reproduces the variability of the observed streamflow, with values closer to 1 indicating better model performance. KGE integrates correlation, bias, and variability components, providing a more comprehensive assessment of the model’s ability to reproduce hydrological dynamics. Vol quantifies the consistency of overall water volume between simulated and observed streamflow, where values near 1 indicate a balanced water budget. A value of 1 mm/day is set for the precipitation/no precipitation threshold in categorical metrics. This choice follows the World Meteorological Organization (WMO) guidelines, where a day with at least 1 mm of precipitation is classified as a “precipitation day.” Lower amounts are typically regarded as trace precipitation, which may have limited hydrological or agricultural impact. Moreover, this threshold is widely adopted in previous studies evaluating satellite or model-based precipitation, ensuring comparability and consistency with existing literature. Formulas of these indices are listed in the Table 2.

**Table 2:** Evaluation criteria.

Evaluation indexes	Equations	Perfect value
Pearson correlation coefficient (CC)	$CC\text{=}\frac{\sum_{i=1}^{n}(S_{i}-\overset{―}{S})(O_{i}-\overset{―}{O})}{\sqrt{\sum_{i=1}^{n}(S_{i}-\overset{―}{S}{)}^{\text{2}}}\sqrt{\sum_{i=1}^{n}(O_{i}-\overset{―}{O}{)}^{\text{2}}}}$	1
Mean Absolute Error (MAE)	$MAE=\frac{\text{1}}{n}\sum_{i=1}^{n}\left|S_{i}-O_{i}\right|$	0
Bias	$Bias=\frac{\text{1}}{N}\sum_{i=1}^{N}(S_{i}-O_{i})$	0
NSE	$NSE=1-\frac{\sum_{i=1}^{N}{\left(Q_{obs,i}-Q_{sim,i}\right)}^{\text{2}}}{\sum_{i=1}^{N}{\left(Q_{obs,i}-{\overset{―}{Q}}_{obs}\right)}^{\text{2}}}$	1
Probability of detection (POD)	$POD=\frac{H}{H+M}$	1
False alarm ratio (FAR)	$FAR=\frac{F}{H+F}$	0
Critical Success Index (CSI)	$CSI=\frac{H}{H+M+F}$	1
KGE	$\begin{array}{c} KGE=1-\sqrt{(CC-1{)}^{\text{2}}+(\beta -1{)}^{\text{2}}+(\gamma -1{)}^{\text{2}}}\\ \beta =\frac{\sigma_{S}}{\sigma_{O}},\gamma =\frac{\mu_{s}}{\mu_{o}} \end{array}$	1
Volume Bias	$Vol=\frac{\sum Q_{sim}}{\sum Q_{obs}}$	1

Note: n denotes the sample size; S_i_ and O_i_ denote ith values of the satellite data and validation data, respectively; S and O denote the average values of S_i_ and O_i_, respectively. H is the number of observed rainfall events that satellite precipitation products (SPPs) correctly detect, M is the number of rainfall events that SPPs miss, F is the number of false detections of SPPs. *β* is the standard deviation ratio of simulated to observed values, and *γ* is the mean ratio of simulated to observed values. $Q_{obs}$ stands for observed streamflow. $Q_{sim}$ stands for simulated streamflow, which is the flow rate predicted by a BTOP model.

## Results

### Accuracy evaluation of satellite precipitation products

To evaluate the detection capability of satellite precipitation datasets, this study compared precipitation estimates from GSMaP and MSWEP at ground station grid points with observed precipitation from ground-based rain gauges. A precipitation event was defined as a day with daily precipitation ≥ 1 mm; otherwise, it was considered a non-precipitation day. Data from 11 ground-based meteorological stations were used, with station indices and names as follows: Station 1 (Bangoin), Station 2 (Nagqu), Station 3 (Jiali), Station 4 (Damxung), Station 5 (Zedang), Station 6 (Lhasa), Station 7 (Mozhugongka), Station 8 (Gongga), Station 9 (Gyaca), Station 10 (Nimu), and Station 11 (Biruo).

The H (Hits), M (Misses), and F (False alarms) values for each station, along with the derived POD (Probability of Detection), FAR (False Alarm Ratio), and CSI (Critical Success Index), are presented in Table 3. Most POD values exceeded 0.9, with an average of 0.9196. The majority of FAR values were below 0.15, averaging 0.1252. Most CSI values were greater than 0.8, with an average of 0.8126, indicating that GSMaP demonstrates strong overall performance in precipitation detection. In terms of POD, all stations exhibited satisfactory detection performance, with Station 6 performing the best. Regarding FAR, Station 4 had the lowest value (0.0762), significantly lower than that of the other stations. CSI values were generally high, with Station 11 showing the lowest CSI (0.7459), and Station 6 showing the highest (0.869), indicating a large performance gap. Overall, Station 6 demonstrated the best comprehensive performance, with POD, FAR, and CSI values of 0.9754, 0.1115, and 0.869, respectively, while Station 11 showed the weakest performance, with POD, FAR, and CSI values of 0.8595, 0.1512, and 0.7453, respectively. Nevertheless, compared with other satellite products such as IMERG and TMPA, GSMaP shows excellent precipitation detection capabilities [55,56].

**Table 3:** Accuracy assessment of GSMaP precipitation estimates.

Station	1	2	3	4	5	6	7	8	9	10	11
H	1822	1713	1749	1854	1755	1904	1846	1797	1713	1799	1633
M	163	238	199	184	140	48	103	102	187	82	267
F	206	240	243	153	296	239	242	292	291	310	291
POD	0.92	0.88	0.90	0.91	0.93	0.98	0.95	0.95	0.90	0.96	0.86
FAR	0.10	0.12	0.12	0.08	0.14	0.11	0.12	0.14	0.15	0.15	0.15
CSI	0.83	0.78	0.80	0.85	0.80	0.87	0.84	0.82	0.78	0.82	0.75

The GSMaP product demonstrates overall strong precipitation detection capabilities over the complex terrain of the TP. It achieves a high average Probability of Detection (POD) of 0.9196, a low average False Alarm Ratio (FAR) of 0.1252, and an average Critical Success Index (CSI) of 0.8126. Compared with multi-source merged products such as MSWEP, GSMaP exhibits higher spatial and temporal consistency in detecting daily precipitation events (≥1 mm) over the plateau. However, its performance in capturing extreme precipitation events and precipitation in transition zones remains to be improved.

In the evaluation of H values, Station 6 performed the best, followed by Station 4, whereas Station 11 performed the worst. This indicates that Stations 6 and 4 had the highest accuracy in identifying precipitation events. Regarding *F* values, Station 4 exhibited the best performance with the lowest false alarm probability, while the other stations had similar *F* values. In terms of *M* values, Station 6 again performed the best, followed by Stations 7, 8, and 10. Station 6 had the lowest *M* value, indicating the fewest missed detections. Conversely, Stations 2 and 11 had the highest *M* values, suggesting a larger number of missed precipitation events. In summary, Stations 6 and 4 exhibited relatively high accuracy in precipitation detection and forecasting, while Station 11 showed weaker performance in both detection and error control, revealing spatial variability in detection capabilities across stations.

The *H*, *M*, and *F* values, as well as the calculated POD, FAR, and CSI metrics for Stations 1–11, are shown in Table 4 for the MSWEP product. All POD values exceeded 0.9, with an average of 0.9301. Most FAR values were below 0.23, with an average of 0.2032. CSI values were mostly above 0.7, averaging 0.7512, suggesting strong overall performance of the MSWEP product in precipitation detection.

**Table 4:** Accuracy assessment of MSWEP precipitation estimates.

Station	1	2	3	4	5	6	7	8	9	10	11
H	1676	1612	1592	1707	1659	1706	1692	1705	1591	1732	1446
M	150	174	117	155	101	91	109	85	133	90	151
F	364	404	481	328	430	393	389	400	466	368	593
POD	0.92	0.90	0.93	0.92	0.94	0.95	0.94	0.95	0.92	0.95	0.91
FAR	0.18	0.20	0.23	0.16	0.21	0.19	0.19	0.19	0.23	0.18	0.29
CSI	0.77	0.74	0.73	0.70	0.76	0.78	0.77	0.78	0.73	0.79	0.66

Compared with GSMaP, MSWEP shows a slightly higher POD but slightly lower FAR and CSI, suggesting that although multi-source fusion can enhance the detection rate of precipitation events, it may also introduce more false alarms due to the inclusion of reanalysis fields and grid-based interpolation. Spatially, Stations 6 and 4 exhibit superior *H*, *M*, and *F* metrics compared to other stations, indicating more stable precipitation characteristics and better data fusion performance in these regions. In contrast, Stations 2 and 11 display higher *M* and *F* values, reflecting the persistence of false alarms and missed detections in MSWEP.

Results indicate that in terms of detection accuracy, Station 10 had the highest *H* value, followed by Stations 4, 6, 7, and 8. Station 11 had the lowest *H* value, implying the weakest detection performance. For false alarms, Station 4 had the lowest *F* value, indicating the best performance, whereas Station 11 had the highest *F* value. Regarding *M* values, Station 2 had the highest number of missed detections, while Station 8 had the lowest, indicating the best performance. These findings highlight the spatial heterogeneity in precipitation detection and forecasting accuracy, with Stations 10 and 4 performing best, while Stations 11 and 2 had higher rates of false and missed detections.

Further analysis of the detection metrics for MSWEP, including POD, FAR, and CSI, confirms that MSWEP demonstrates strong overall detection capability in the Lhasa River Basin. Among all stations, Station 10 achieved the highest POD (0.9506), suggesting the best detection effectiveness, while Station 11 had the lowest POD (0.9054). In terms of false alarm rate, all stations had relatively low FAR values, with Station 4 having the lowest (0.1612) and Station 11 the highest (0.2908), approximately twice that of Station 4. CSI values also reflect this trend; all stations, except Station 11 (CSI = 0.6603), had values above 0.72, with Station 10 reaching the highest CSI of 0.7909. Overall, Station 10 demonstrated the best comprehensive detection performance, while Station 11 performed the worst. Despite spatial variations across stations, MSWEP outperforms other products such as IMERG and TMPA in the Lhasa River Basin, showing high application potential.

### Spatial accuracy comparison of satellite precipitation products

**Fig 3:**
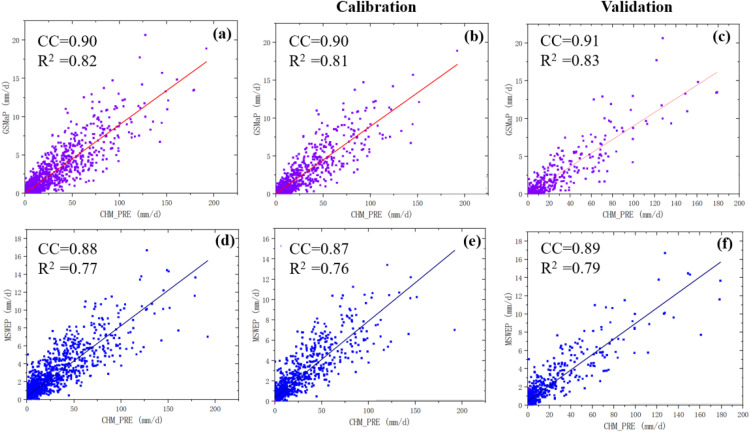
Comparison of satellite-derived versus observed precipitation scatter plots during calibration and validation periods. **(a)**–(c) show scatter plots for GSMaP against gauge observations during the calibration period, calibration subset, and validation period, respectively, while **(d)**–(f) present the corresponding results for MSWEP.

To evaluate the accuracy and applicability of the two satellite precipitation products in the Lhasa River Basin, scatter plots were generated using daily precipitation time series data from meteorological stations and the two datasets for the period from January 1, 2010, to December 31, 2015, as shown in Fig 3 (a) – (f). Regression analyses were conducted between each precipitation product and the observed values, from which the fitted equations, coefficient of determination (R^2^), and correlation coefficient (CC) were derived.

This study assessed the applicability of the GSMaP and MSWEP satellite precipitation products in the Lhasa River Basin and analyzed their precipitation simulation accuracy. The results show that GSMaP exhibits CCs and R^2^ values ranging between 0.90–0.91 and 0.81–0.83, respectively.

In comparison, the MSWEP product had CCs and R^2^ values ranging from 0.87–0.91 and 0.76–0.83, respectively. The best performance occurred during the calibration period (January 1, 2014, to December 31, 2015), while the poorest was during the validation period (January 1, 2010, to December 31, 2013). Although MSWEP performed slightly less accurately than GSMaP, its overall performance remained acceptable. Moreover, the fitted equations of the two precipitation products were generally similar. The simulation accuracy of the fitted slopes was high (with a variance of approximately 0.01), while the fit of the intercepts was relatively weaker but still acceptable (values ranging from 0.025 to 0.046).

By comprehensively comparing the applicability of GSMaP and MSWEP in the Lhasa River Basin, it can be concluded that GSMaP outperforms MSWEP in overall precipitation simulation accuracy, with better fitting performance, particularly during the calibration period and across the full study period. Although both satellite products show good applicability, GSMaP demonstrates stronger precipitation estimation capabilities in the Lhasa River Basin, making it a valuable data source for hydrometeorological studies in the region.

**Fig 4:**
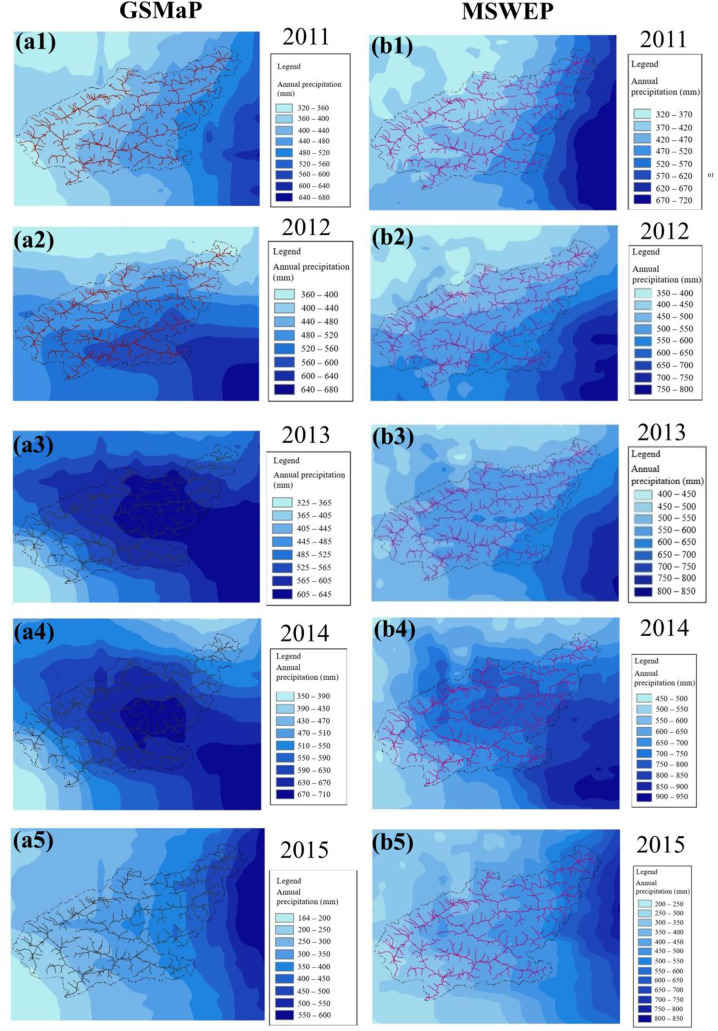
Comparison of mean annual precipitation spatial patterns between GSMaP and MSWEP satellite products. (a1)–(a5) show the spatial distribution of annual precipitation derived from GSMaP for the years 2011–2015, while (b1)–(b5) present the corresponding results from MSWEP for the same years.

To further explore spatial-scale characteristics, annual mean precipitation distribution maps over the BTOP model calculation region were generated based on runoff time series derived from the two precipitation products (Fig 4 (a1) – (b5)). Regarding the spatial distribution pattern of precipitation, both GSMaP and MSWEP show a similar precipitation gradient trend—increasing from west to east. However, there are significant differences in the finer details of the spatial distribution.

A comparative analysis of the interannual spatial distribution patterns of the two precipitation products, GSMaP and MSWEP, reveals both consistencies and discrepancies in their representation of precipitation amounts and centers. In 2011, although both products identified the precipitation center at similar locations and estimated comparable total amounts, significant differences were observed in the spatial distribution patterns and the gradients of precipitation intensity. These discrepancies may be attributed to differences in the products’ precipitation intensity resolution, grid scales, or their ability to resolve precipitation structure.

In 2014, the spatial distribution of precipitation was generally similar between the two products; however, substantial deviations were noted in the absolute precipitation estimates. GSMaP estimated a maximum precipitation amount of approximately 700 mm at the center, whereas MSWEP exceeded 900 mm. This suggests the presence of systematic errors in the products’ ability to capture extreme precipitation events, possibly stemming from issues such as radiometric calibration of satellite sensors, inconsistencies in retrieval algorithms, or differences in ground-based observational data used for correction.

For 2012 and 2013, the two products not only displayed considerable differences in precipitation magnitude but also showed spatial shifts in the locations of precipitation centers. Nevertheless, both products exhibited a similar gradient pattern, with precipitation increasing from west to east across the basin. Despite this overall consistency, notable differences emerged in the finer details of spatial distribution. MSWEP showed more concentrated and intense precipitation in the southeastern region, while GSMaP exhibited a smoother distribution, with relatively higher precipitation in central and western areas. In general, MSWEP tended to report higher precipitation magnitudes than GSMaP, particularly in the eastern and southeastern parts of the basin, where heavy rainfall was more prominent in MSWEP’s estimates. In contrast, GSMaP showed lower precipitation in the northwestern areas, whereas MSWEP estimated comparatively higher values. Both products captured the regional east–west gradient, with higher precipitation in the east and lower in the west.

These results suggest that, while both precipitation products are broadly consistent in capturing the regional spatiotemporal characteristics of precipitation, discrepancies remain in terms of magnitude and localized features. These differences may arise from the nature of the data sources used. MSWEP integrates multiple data sources such as reanalysis, ground-based observations, and satellite data, which enhances its data assimilation capabilities and allows it to better represent extreme precipitation events, particularly in regions with high precipitation. In contrast, GSMaP relies primarily on satellite-based remote sensing, and its algorithm may lead to underestimation in complex terrain or low-precipitation regions. Consequently, MSWEP may offer more accurate estimates in high-precipitation zones, while GSMaP could underestimate precipitation in localized areas. Therefore, when using GSMaP in hydrological studies, post-processing steps such as bias correction or data fusion may be necessary to improve data reliability. If the study objective involves the analysis of extreme precipitation events or detailed spatiotemporal distribution characteristics, MSWEP may be a more suitable choice.

### Evaluation of hydrological model performance

In this study, satellite-based and ground-observed precipitation data from January 1, 2010, to December 31, 2015, were used to calibrate and validate the BTOP hydrological model. The period from January 1, 2010, to December 31, 2013, was designated as the calibration period, while the period from January 1, 2014, to December 31, 2015, was used for validation. The Nash–Sutcliffe efficiency coefficient (NSE) and Kligwas calculated for both precipitation products during the calibration and validation periods. In addition, the volumetric ratio (Vol) was computed to assess the accuracy of streamflow simulations. The NSE values obtained are presented in Table 5.

**Table 5:** Performance metrics of the two precipitation products during calibration and validation periods.

metrics	GSMaP calibration	GSMaP validation	Uncertainty (%)	MSWEP calibration	MSWEP validation	Uncertainty (%)
*NSE*	0.793	0.541	−31.8	0.826	0.431	−47.8
*KGE*	0.847	0.635	−25.1	0.769	0.572	−25.6
*Vol*	0.995	0.902	−9.3	0.984	0.720	−26.8

As shown in Table 5, the NSE values during the calibration period are significantly higher than those in the validation period, indicating that the model performs better during calibration. Specifically, the GSMaP product yielded an NSE of 0.793 during calibration, while the MSWEP product achieved a higher NSE of 0.826, suggesting that MSWEP provided better performance in simulating runoff during that period. However, during the validation period, GSMaP exhibited superior performance, with a higher NSE compared to MSWEP. Regarding the volumetric ratio (Vol) between simulated and observed streamflow, the GSMaP product achieved 0.995 and the MSWEP product 0.984 during the calibration period, both indicating excellent performance. However, in the validation period, the Vol values decreased to 0.902 for GSMaP and 0.720 for MSWEP, with the latter showing a notably lower performance than in the calibration period. Overall, both satellite precipitation products maintain Vol values above 0.70, indicating relatively small discrepancies between simulated and observed streamflow and generally satisfactory simulation results. In terms of the water balance index, all values for GSMaP (calibration and validation) and MSWEP (calibration and validation) exceeded 0.94, reflecting high reliability of both products in maintaining water balance.

To further quantify the uncertainty associated with the sparse rain gauge network, we compared the performance degradation between the calibration and validation periods for both precipitation products. The relative changes in NSE, KGE, and Vol, provide a measure of temporal robustness. As shown in Table 5, GSMaP exhibits substantially smaller uncertainty ranges, with decreases of 31.8% in NSE, 25.1% in KGE, and only 9.3% in volumetric accuracy. In contrast, MSWEP shows much larger reductions of 47.8%, 25.6%, and 26.8%, respectively. These results indicate that MSWEP suffers from stronger temporal non-stationarity and larger fluctuations in error structure, whereas GSMaP demonstrates notably better stability and transferability across hydrological periods. Therefore, although both products show reduced performance during validation, GSMaP provides more consistent forcing inputs for hydrological modeling in this data-scarce alpine basin.

To further explore the temporal performance of the BTOP model using GSMaP and MSWEP precipitation inputs, daily runoff time series were generated and are illustrated in –. In these figures, “obs” denotes observed streamflow, “BTOP” refers to model-simulated streamflow, and “P” indicates observed precipitation. The results show that both products yielded satisfactory simulations during the calibration period, although performance deteriorated somewhat during the validation period.

**Fig 5:**
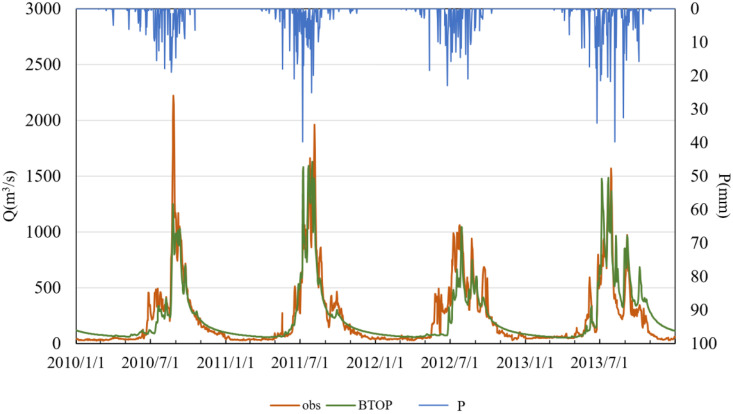
Simulated versus observed daily runoff driven by GSMaP data in the calibration period.

**Fig 6:**
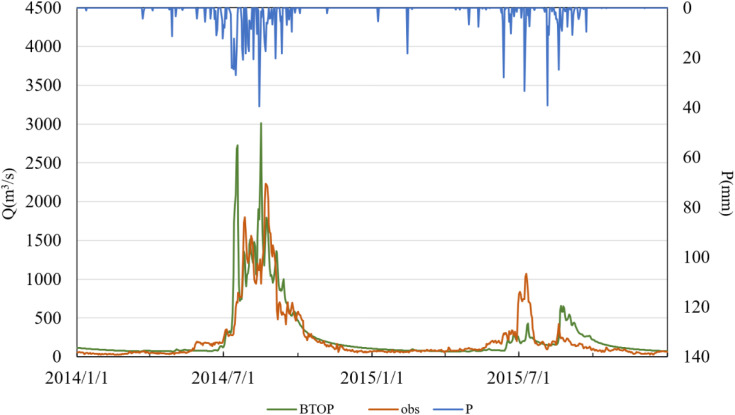
Simulated versus observed daily runoff driven by GSMaP data in the validation period.

**Fig 7:**
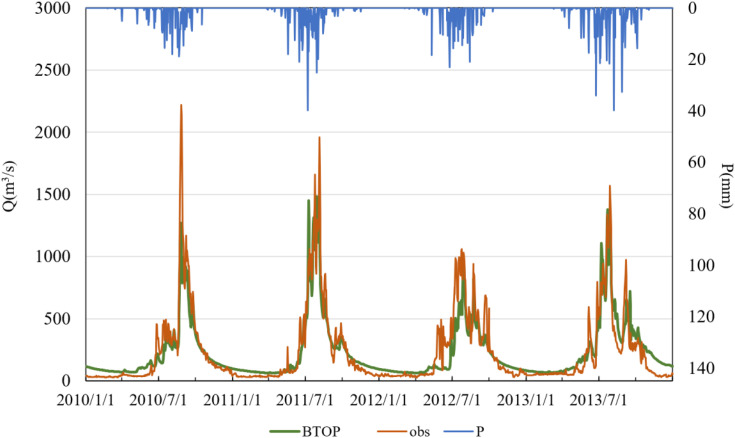
Simulated versus observed daily runoff driven by MSWEP data in the calibration period.

**Fig 8:**
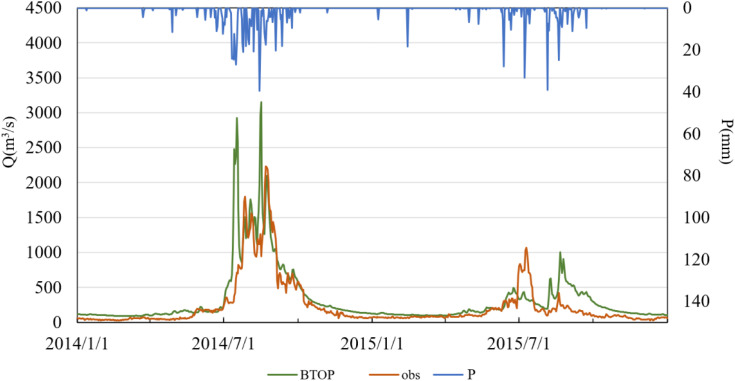
Simulated versus observed daily runoff driven by MSWEP data in the validation period.

The simulation results based on the GSMaP satellite precipitation product demonstrate that the BTOP model effectively captures the seasonal and interannual variability of runoff in the Lhasa River Basin during the calibration period (2010–2013). The simulated hydrographs closely match the observed discharge in both dry and wet seasons in terms of timing and magnitude. The Nash–Sutcliffe Efficiency (NSE) coefficients all exceed 0.50, indicating strong model performance in reproducing the overall runoff dynamics. However, in July 2014, the simulated peak discharge was significantly lower than the observed value, reflecting an underestimation of the hydrological response to extreme precipitation events. During the validation period (2014–2015), GSMaP-driven simulations exhibited an overestimation of peak flow in July 2014, with the simulated peak occurring approximately 1–2 days earlier than the observed peak. This suggests that limitations in the temporal resolution of the precipitation data or model parameterization may affect the dynamic response to heavy rainfall events. Nevertheless, the NSE values during the validation period remained within an acceptable range, confirming the applicability of GSMaP for hydrological modeling in complex terrain.

The performance of MSWEP precipitation data during the calibration period (2010–2013) was also satisfactory, with minimal phase shifts between simulated and observed flows. The model reproduced flow fluctuations well, particularly during periods of moderate discharge. However, the simulation accuracy declined markedly during the validation period (2014–2015), with the NSE dropping below 0.50. Specifically, in July 2014, the simulated peak discharge exceeded observations, and the peak timing in July 2014 and 2015 was either advanced or delayed by 2–3 days, respectively. These discrepancies may be attributed to the sparse distribution of ground meteorological stations in the Lhasa River Basin, which limits the spatial representativeness of the MSWEP product. In particular, satellite-derived estimates may struggle to capture localized intense precipitation events, thereby affecting the temporal and spatial accuracy of runoff simulations.

Comparative analysis of the two precipitation products indicates that GSMaP exhibits greater overall stability than MSWEP during both calibration and validation periods, with consistently higher NSE values and smaller timing deviations. This suggests that GSMaP provides a more reliable characterization of the spatiotemporal distribution of precipitation in the Lhasa River Basin. While MSWEP performed well during the calibration period, its degraded performance in the validation period reveals limitations in data-sparse regions. Overall, GSMaP appears to be more suitable for high-resolution hydrological simulations in this region. In contrast, MSWEP may require the integration of ground observations to enhance its spatial representativeness. Both products exhibited discrepancies in peak flow response to extreme precipitation events, indicating a need for further optimization of model parameters or the development of multi-source precipitation fusion approaches to improve simulation accuracy.

## Discussion

To further contextualize the performance of satellite-based precipitation products in this study, we compared our findings with similar studies conducted across the Tibetan Plateau (TP) and adjacent regions. Liu et al. [57] evaluated TRMM, CPC, CMFD, and ERA5-Land and found that CMFD performed best on daily and monthly scales, while TRMM and ERA5-Land performed worse in high-altitude and arid areas. In contrast, our results show that GSMaP outperforms MSWEP in the Lhasa River Basin in terms of CSI, CC, and NSE, indicating higher reliability for both precipitation estimation and runoff simulation. Wang et al. [58] reported that model calibration can partially offset input errors but becomes insufficient once precipitation errors exceed a certain threshold, while Zhang et al. [59] found that GPM performs well during high-flow periods despite negative biases. These studies, together with our results, highlight that careful selection and evaluation of SPPs are critical in mountainous regions and that GSMaP provides a clear advantage for hydrological applications in high-altitude, data-scarce basins such as the central TP. The relatively good performance of MSWEP during calibration but its deterioration during validation also suggests potential overfitting of model parameters to error structures specific to the calibration period, emphasizing the need for bias correction, long-term cross-validation, and multi-source precipitation fusion.

From a process perspective, the moderate validation performance of the GSMaP-driven BTOP model (NSE = 0.54) and the contrasting underestimation and overestimation of peak flows can be explained by the combined effects of satellite precipitation errors and model structural limitations. GSMaP exhibits year- and season-dependent biases, including underestimation of intense rainfall and rain–snow phase misclassification near 0 °C, and these error characteristics differ between the 2010–2013 calibration period and the 2014–2015 validation period. During the 2010 flood season, GSMaP underestimates the intensity and duration of several multi-day rainfall events over the upper basin, leading to insufficient soil moisture build-up in BTOP and muted simulated peaks, even when the timing of the rising limb is reasonable. In contrast, the 2014 flood season is dominated by localized, short-duration convective storms that are partially smoothed and spatially displaced by the 0.1° daily GSMaP and MSWEP grids; when projected over an overly large effective contributing area in the model, these storms can trigger excessive runoff and produce simulated peaks that are higher and slightly earlier than observed. Moreover, BTOP uses a single parameter set for the entire simulation period; parameters calibrated under predominantly snowmelt- and stratiform-rain-dominated conditions may be too responsive to more intense convective rainfall, further amplifying peak overestimation during the validation period. Overall, these findings indicate that peak-flow errors arise from non-linear interactions between precipitation timing/intensity errors and model structure, rather than from a simple bias in seasonal precipitation totals, and they support the use of season-specific bias correction and, where possible, regime-dependent parameterization in high-altitude basins.

The BTOP model has been applied in diverse regions worldwide and shows good adaptability in both humid and data-scarce basins, and in this study it effectively captures the main hydrological processes of the glacier-influenced Lhasa River Basin. Nevertheless, as a semi-distributed model, BTOP simplifies spatial heterogeneity by grouping similar terrain units and may overlook sub-grid variability in glacier dynamics, permafrost, and localized land-use changes, as well as uncertainties in snow and glacier melt simulation under limited observations. Future work should therefore focus on integrating high-resolution remote sensing and improving the representation of cryospheric processes, while also incorporating bias-correction techniques and advanced data-fusion methods, including machine learning, to further reduce precipitation uncertainties. The use of next-generation high-resolution datasets (e.g., GPM IMERG) offers new opportunities for improving precipitation estimation in high-altitude basins. Strengthening the practical application of GSMaP-driven hydrological simulations—particularly for flood forecasting and early warning in Tibet—and integrating dense ground-based observations and numerical model outputs to develop rainfall-intensity- and season-dependent bias-correction schemes will be crucial for enhancing the stability and transferability of hydrological simulations in the plateau region [60,61]. Future research should integrate dense ground-based observations and numerical model outputs to develop machine learning-based bias correction models tailored to different rainfall intensities and time periods. Additionally, incorporating combined microwave-infrared satellite retrieval algorithms may further reduce the false alarm rate while maintaining high detection capability, thereby improving the CSI and providing more reliable data support for water resources monitoring and management in the plateau region.

## Conclusions

This study evaluates the applicability of the GSMaP and MSWEP satellite precipitation products in the Lhasa River Basin, in combination with the BTOP hydrological model for runoff simulation. The main conclusions are as follows:

(1) Both GSMaP and MSWEP demonstrate satisfactory precipitation estimation capabilities in the Lhasa River Basin. Notably, GSMaP exhibits superior overall performance, with its mean Critical Success Index (CSI = 0.8126) significantly exceeding that of MSWEP (CSI = 0.7521). Moreover, GSMaP maintains higher estimation stability across all evaluated stations. Spatially, both products consistently exhibit high detection accuracy (CSI > 0.66). The Lhasa Station yields optimal results (GSMaP CSI = 0.8690), while the Biruo Station shows relatively inferior performance.
(2) Strong correlations (CC > 0.8) between both products and ground observations are observed during the flood season, whereas correlations markedly diminish in the dry season (December–February). GSMaP outperforms MSWEP across all error indices, particularly demonstrating lower systematic bias in temporal analysis. Linear regression further confirms GSMaP’s superiority, with higher R² values during the entire period (0.82), validation phase (0.81), and calibration phase (0.83) compared to MSWEP (0.77, 0.76, and 0.79, respectively).
(3) The BTOP model driven by satellite precipitation data effectively replicates the runoff processes in the Lhasa River Basin. Simulations forced by GSMaP achieve higher Nash-Sutcliffe efficiency coefficients (NSE) than those using MSWEP, with notably better performance during the calibration period (NSE > 0.75) than the validation period. Although slight overestimation (2014 flood season) and underestimation (2010 flood season) occur, the overall simulations capture the basin’s hydrological dynamics robustly, validating the model’s applicability in high-altitude regions.

## References

[1] Smith C, Baker JCA, Spracklen DV. Tropical deforestation causes large reductions in observed precipitation. Nature. 2023;615(7951):270–5. doi: 10.1038/s41586-022-05690-1 36859548 PMC9995269

[2] Liang Y, Zhao H, Yuan Z, Wei D, Wang X. Ecological Restoration Projects Adapt Response of Net Primary Productivity of Alpine Grasslands to Climate Change across the Tibetan Plateau. Remote Sens. 2024;16(23):4444. doi: 10.3390/rs16234444

[3] Wang X, Wu B, Zhou G, Wang T, Meng F, Zhou L, et al. How a vast digital twin of the Yangtze River could prevent flooding in China. Nature. 2025;639(8054):303–5. doi: 10.1038/d41586-025-00720-0 40069418

[4] Bevacqua E, Zappa G, Lehner F, Zscheischler J. Precipitation trends determine future occurrences of compound hot–dry events. Nat Clim Chang. 2022;12(4):350–5. doi: 10.1038/s41558-022-01309-5

[5] Zhang W, Furtado K, Zhou T, Wu P, Chen X. Constraining extreme precipitation projections using past precipitation variability. Nat Commun. 2022;13(1):6319. doi: 10.1038/s41467-022-34006-0 36329032 PMC9633619

[6] Zhou L, Koike T, Takeuchi K, Rasmy M, Onuma K, Ito H, et al. A study on availability of ground observations and its impacts on bias correction of satellite precipitation products and hydrologic simulation efficiency. J Hydrol. 2022;610:127595. doi: 10.1016/j.jhydrol.2022.127595

[7] Yan L, Kong L, Wang L, Zhang L, Hu J, Ouyang Z. Grass-livestock balance under the joint influences of climate change, human activities and ecological protection on Tibetan Plateau. Ecol Indicat. 2024;162:112040. doi: 10.1016/j.ecolind.2024.112040

[8] Guo R, Fan X, Zhou H, Liu Y. Multi-Sensor Precipitation Estimation from Space: Data Sources, Methods and Validation. Remote Sens. 2024;16(24):4753. doi: 10.3390/rs16244753

[9] Du J, Yu X, Zhou L. Less concentrated precipitation and more extreme events over the three river headwaters region of the Tibetan Plateau in a warming climate. Atmos Res. 2024;:107311.

[10] Smith AJ, Baeck LM, Miller JA. Rainfall frequency analysis based on long‐term high‐resolution radar rainfall fields: spatial heterogeneities and temporal nonstationarities. Water Resourc Res. 2024;60(3).

[11] Liu Z. Comprehensive Evaluation of High-Resolution Satellite Precipitation Products over the Qinghai–Tibetan Plateau Using the New Ground Observation Network. Remote Sens. 2023;15(13):3381. doi: 10.3390/rs15133381

[12] Sun W, Zhang E, Ni Z, Liu Y, Meng X, Han W, et al. Abrupt middle to late Holocene hydroclimate fluctuations on the northwestern Qinghai-Tibetan Plateau inferred from lacustrine carbonate isotopes. Catena. 2024;239:107908. doi: 10.1016/j.catena.2024.107908

[13] Miao C, Gou J, Hu J. Impacts of Different Satellite‐Based Precipitation Signature Errors on Hydrological Modeling Performance Across China. Earth’s Future. 2024;12(11):e2024EF004954.

[14] Katsanos D, Retalis A, Kalogiros J, Psiloglou BE, Roukounakis N, Anagnostou M. Performance Evaluation of Satellite Precipitation Products During Extreme Events—The Case of the Medicane Daniel in Thessaly, Greece. Remote Sens. 2024;16(22):4216. doi: 10.3390/rs16224216

[15] Lv P, Wu G. The Performance of GPM IMERG Product Validated on Hourly Observations over Land Areas of Northern Hemisphere. Remote Sens. 2024;16(22):4334. doi: 10.3390/rs16224334

[16] Zheng G, Zhang N, Zhang L. A downscaling method of TRMM satellite precipitation based on geographically neural network weighted regression: A case study in Sichuan province, China. Atmosphere. 2024;15(7):792.

[17] Gan Y, Li Y, Wang L, Zhao L, Fan L, Xu H, et al. Machine-learning downscaling of GPM satellite precipitation products in mountainous regions: A case study in Chongqing. Atmos Res. 2024;311:107698. doi: 10.1016/j.atmosres.2024.107698

[18] Yue J, Zhou L, Du J. Runoff simulation in data-scarce alpine regions: comparative analysis based on LSTM and physically based models. Water. 2024;16(2161):2161.

[19] Keikhosravi-Kiany MS, Balling RC Jr. Evaluation of GPM IMERG Early, Late, and Final Run in Representing Extreme Rainfall Indices in Southwestern Iran. Remote Sens. 2024;16(15):2779. doi: 10.3390/rs16152779

[20] Li W, Kang Y, Li L, Gao R, Shu Z, Song S. Comprehensive assessment of five near-real-time satellite precipitation products in the Lower Yangtze River Basin and the Lixiahe region, China: Dual perspectives from time series and extreme events. Atmos Res. 2024;308:107520. doi: 10.1016/j.atmosres.2024.107520

[21] Yang S, Surratt M, Whitcomb RT, et al. Evaluation of IMERG and GSMaP for Tropical Cyclone Applications. Geophys Res Lett. 2024;51(4).

[22] Tang X, Zhang J, Wang G, Ruben GB, Bao Z, Liu Y, et al. Error Correction of Multi-Source Weighted-Ensemble Precipitation (MSWEP) over the Lancang-Mekong River Basin. Remote Sens. 2021;13(2):312. doi: 10.3390/rs13020312

[23] Li L, Wang Y, Wang L, Hu Q, Zhu Z, Li L, et al. Spatio-temporal accuracy evaluation of MSWEP daily precipitation over the Huaihe River Basin, China: A comparison study with representative satellite- and reanalysis-based products. J Geogr Sci. 2022;32(11):2271–90. doi: 10.1007/s11442-022-2047-9

[24] Xiang Y, Chen J, Li L, Peng T, Yin Z. Evaluation of Eight Global Precipitation Datasets in Hydrological Modeling. Remote Sens. 2021;13(14):2831. doi: 10.3390/rs13142831

[25] Zhu H, Chen K, Hu S, Liu J, Shi H, Wei G, et al. Using the Global Navigation Satellite System and Precipitation Data to Establish the Propagation Characteristics of Meteorological and Hydrological Drought in Yunnan, China. Water Resourc Res. 2023;59(4). doi: 10.1029/2022wr033126

[26] Dong N, Xu X, Cai W, Zhao T, Sun C. Comprehensive effects of interdecadal change of sea surface temperature increase in the Indo-Pacific Ocean on the warming-wetting of the Qinghai-Tibet Plateau. Sci Rep. 2022;12(1):22306. doi: 10.1038/s41598-022-26465-8 36566284 PMC9789985

[27] Li J, Wu L, Liu J, Wang X, Xue W. DFMM-Precip: Deep Fusion of Multi-Modal Data for Accurate Precipitation Forecasting. Water. 2024;16(24):3702. doi: 10.3390/w16243702

[28] Kang X, Dong J, Crow WT, Wei L, Zhang H. The Conditional Bias of Extreme Precipitation in Multi‐Source Merged Data Sets. Geophys Res Lett. 2024;51(22). doi: 10.1029/2024gl111378

[29] Li R, Liu C, Tang Y, Niu C, Fan Y, Luo Q, et al. Study on Runoff Simulation with Multi-source Precipitation Information Fusion Based on Multi-model Ensemble. Water Resour Manage. 2024;38(15):6139–55. doi: 10.1007/s11269-024-03949-y

[30] Shi J, Zhang J, Bao Z, Parajka J, Wang G, Liu C, et al. A novel error decomposition and fusion framework for daily precipitation estimation based on near-real-time satellite precipitation product and gauge observations. J Hydrol. 2024;640:131715. doi: 10.1016/j.jhydrol.2024.131715

[31] Zhou Z, Zhou F, Zhang M, Lei B, Ma Z. Effect of increasing rainfall on the thermal—moisture dynamics of permafrost active layer in the central Qinghai—Tibet Plateau. J Mt Sci. 2021;18(11):2929–45. doi: 10.1007/s11629-021-6707-5

[32] Liu S, Zhou T, Jiang J, Zou L, Zhang L, Zhang W, et al. Contributions of Stationary and Transient Water Vapor Transports to the Extreme Precipitation Changes Over the Tibetan Plateau. JGR Atmos. 2024;129(22). doi: 10.1029/2024jd040966

[33] Feng Y, Qi Y, Zhao Z, Li D. Can Satellite or Reanalysis Precipitation Products Depict the Location and Intensity of Rainfall at Flash Flood Scale Over the Eastern Mountainous Area of the Tibetan Plateau? Water Resourc Res. 2024;60(11). doi: 10.1029/2024wr037381

[34] Li L, Dong X, Ma Y. Relationship between Tibetan Plateau Surface Heat Fluxes and Daily Heavy Precipitation in the Middle and Lower Yangtze River Basins (1980–2022). Remote Sens. 2024;16(20):3779.

[35] Luo L, Zhao Y, Duan Y, Dan Z, Acharya S, Jimi G, et al. Relationships between Precipitation and Elevation in the Southeastern Tibetan Plateau during the Active Phase of the Indian Monsoon. Water. 2024;16(18):2700. doi: 10.3390/w16182700

[36] Yin Y, Wang J, Leng G, Zhao J, Wang L, Ma W. Future potential distribution and expansion trends of highland barley under climate change in the Qinghai-Tibet plateau (QTP). Ecol Indicat. 2022;136:108702. doi: 10.1016/j.ecolind.2022.108702

[37] Zhou C, Zhou L, Du J, Yue J, Ao T. Accuracy evaluation and comparison of GSMaP series for retrieving precipitation on the eastern edge of the Qinghai-Tibet Plateau. J Hydrol. 2024;56:102017. doi: 10.1016/j.ejrh.2024.102017

[38] Liu L, Ao T, Zhou L, Takeuchi K, Gusyev M, Zhang X, et al. Comprehensive evaluation of parameter importance and optimization based on the integrated sensitivity analysis system: A case study of the BTOP model in the upper Min River Basin, China. J Hydrol. 2022;610:127819. doi: 10.1016/j.jhydrol.2022.127819

[39] Lu H-L, Qiu J, Li M-J, Zuo H-M, Li J-L, Hu BX, et al. Temporal and spatial variations in the sub-daily precipitation structure over the Qinghai-Tibet Plateau (QTP). Sci Total Environ. 2024;915:170153. doi: 10.1016/j.scitotenv.2024.170153 38232821

[40] Li D, Tian P, Luo H, Hu T, Dong B, Cui Y, et al. Impacts of land use and land cover changes on regional climate in the Lhasa River basin, Tibetan Plateau. Sci Total Environ. 2020;742:140570. doi: 10.1016/j.scitotenv.2020.140570 32721730

[41] Tian P, Lu H, Feng W, Guan Y, Xue Y. Large decrease in streamflow and sediment load of Qinghai–Tibetan Plateau driven by future climate change: A case study in Lhasa River Basin. CATENA. 2020;187:104340. doi: 10.1016/j.catena.2019.104340

[42] Mega T, et al. Gauge adjusted global satellite mapping of precipitation (GSMaP_Gauge). 2014 XXXIth URSI General Assembly and Scientific Symposium (URSI GASS). IEEE; 2014. p. 1–4.

[43] Bagtasa G. Assessment of Tropical Cyclone Rainfall from GSMaP and GPM Products and Their Application to Analog Forecasting in the Philippines. Atmosphere. 2022;13(9):1398. doi: 10.3390/atmos13091398

[44] Abbas H, Song W, Wang Y, et al. Validation of CRU TS v4.08, ERA5-Land, IMERG v07B, and MSWEP v2.8 Precipitation Estimates Against Observed Values over Pakistan. Remote Sens. 2024;16(24):4803.

[45] Takeuchi K, Hapuarachchi P, Zhou M, Ishidaira H, Magome J. A BTOP model to extend TOPMODEL for distributed hydrological simulation of large basins. Hydrol Process. 2007;22(17):3236–51. doi: 10.1002/hyp.6910

[46] Gusyev M, Magome J, Kiem A, et al. The BTOP model with supplementary tools user manual. 2017.

[47] Beven KJ, Kirkby MJ. A physically based, variable contributing area model of basin hydrology. Hydrol Sci Bull. 1979;1(24):43–69.

[48] Tianqi A, Takeuchi K, Ishidaira H, Yoshitani J, Fukami K. Development and application of a new algorithm for automated pit removal for grid DEMs. Hydrol Sci J. 2003;48(6):985–97. doi: 10.1623/hysj.48.6.985.51423

[49] Nimai S, Ren Y, Ao T. Enhancing runoff simulation using BTOP-LSTM hybrid model in the Shinano River basin. Water. 2023;15(21).

[50] Zhu Y, Liu L, Qin F, Zhou L, Zhang X, Chen T, et al. Application of the Regression-Augmented Regionalization Approach for BTOP Model in Ungauged Basins. Water. 2021;13(16):2294. doi: 10.3390/w13162294

[51] Duan QY, Gupta VK, Sorooshian S. Shuffled complex evolution approach for effective and efficient global minimization. J Optim Theory Appl. 1993;76(3):501–21. doi: 10.1007/bf00939380

[52] Gusyev MA, Magome J, Takeuchi K. Development and application of BTOP model for global-scale distributed hydrological simulation. J Japan Soc Civ Eng Ser B1 (Hydraulic Eng). 2017;73(4):I_625-I_630.

[53] Zhou Z, Guo B, Xing W, Zhou J, Xu F, Xu Y. Comprehensive evaluation of latest GPM era IMERG and GSMaP precipitation products over mainland China. Atmos Res. 2020;246:105132. doi: 10.1016/j.atmosres.2020.105132

[54] Deng P, Zhang M, Guo H, Xu C, Bing J, Jia J. Error analysis and correction of the daily GSMaP products over Hanjiang River Basin of China. Atmos Res. 2018;214:121–34. doi: 10.1016/j.atmosres.2018.07.022

[55] Ma Q, Li Y, Feng H, Yu Q, Zou Y, Liu F, et al. Performance evaluation and correction of precipitation data using the 20-year IMERG and TMPA precipitation products in diverse subregions of China. Atmos Res. 2021;249:105304. doi: 10.1016/j.atmosres.2020.105304

[56] Nepal B, Shrestha D, Sharma S, Shrestha MS, Aryal D, Shrestha N. Assessment of GPM-Era Satellite Products’ (IMERG and GSMaP) Ability to Detect Precipitation Extremes over Mountainous Country Nepal. Atmosphere. 2021;12(2):254. doi: 10.3390/atmos12020254

[57] Liu J, Zhou Y, Lu F, Yu Y, Yan D, Hu Y, et al. Evaluating satellite‐ and reanalysis‐based precipitation products over the Qinghai‐Tibetan Plateau in the perspective of a new error‐index system. Intl J Climatol. 2023;43(5):2200–19. doi: 10.1002/joc.7970

[58] Wang Y, Yang T, Li Y, et al. Evaluation of five satellite-based precipitation products in two gauge-scarce basins on the Tibetan Plateau. Remote Sens. 2018;10(8):1316.

[59] Zhang Y, Ju Q, Zhang L, Xu C-Y, Lai X. Evaluation and Hydrological Application of Four Gridded Precipitation Datasets over a Large Southeastern Tibetan Plateau Basin. Remote Sens. 2022;14(12):2936. doi: 10.3390/rs14122936

[60] Arora M, Sahoo S, Bhatt CM, Litoria PK, Pateriya B. Rapid flood inundation mapping and impact assessment using Sentinel-1 SAR data over Ghaggar River basin of Punjab, India. J Earth Syst Sci. 2023;132(4). doi: 10.1007/s12040-023-02199-7

[61] Arora M, Dixit M, Pateriya B. Assessment of Water Storage Changes Using Satellite Gravimetry and GLDAS Observations over a Part of Indus Basin, India. Water Conserv Sci Eng. 2022;7(4):623–45. doi: 10.1007/s41101-022-00169-6

